# Supplementary material for: Stamped production of single-crystal hexagonal boron nitride monolayers on various insulating substrates
Source: Nat Commun. 2023 Oct 12;14:6421. doi: 10.1038/s41467-023-42270-x (PMC10570391; doi:10.1038/s41467-023-42270-x)
Supplement: Supplementary file 1 — Supplementary Information [file 41467_2023_42270_MOESM1_ESM.pdf]

Supplementary information for

Stamped production of single-crystal hexagonal boron nitride monolayers on various insulating substrates

Fankai Zeng[#], Ran Wang[#], Wenya Wei[#], Zuo Feng[#], Quanlin Guo, Yunlong Ren, Guoliang Cui, Dingxin Zou, Zhensheng Zhang, Song Liu, Kehai Liu, Ying Fu, Jinzong Kou, Li Wang, Xu Zhou, Zhilie Tang, Feng Ding, Dapeng Yu, Kaihui Liu*, and Xiaozhi Xu*

The supplementary information includes:

Supplementary Fig. 1-15

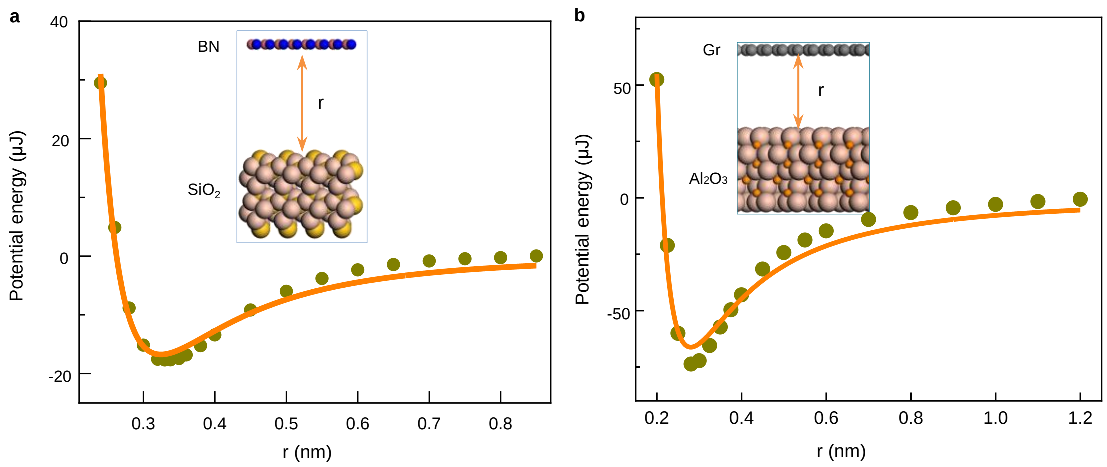

Supplementary Fig. 1 | The vdW interaction of BN on SiO_2 and graphene on Al_2O_3. Plot and fit of the potential energy as a function of the distance between BN and SiO_2 **(a)** and graphene and Al_2O_3 **(b)**.

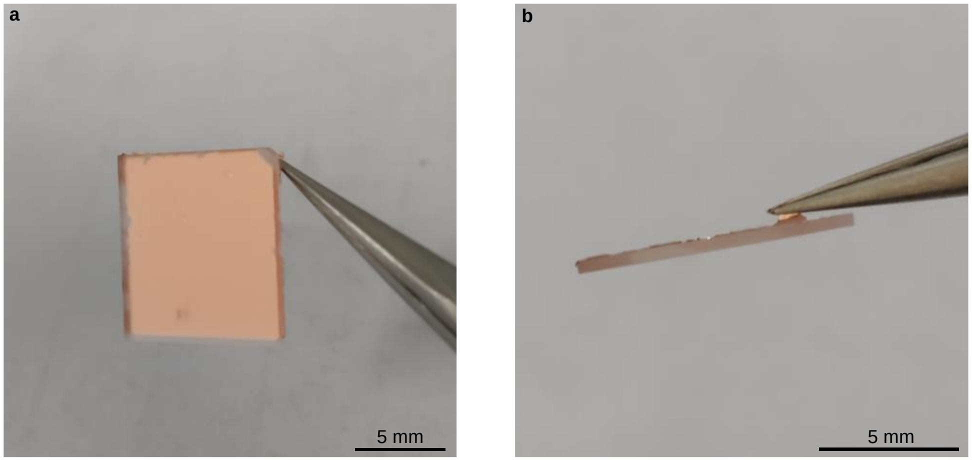

Supplementary Fig. 2 | Optical image of the SiO_2/BN/Cu sandwiched structure. The top and side view of the SiO_2/BN/Cu sandwiched structure are shown in **(a)** and **(b)**, respectively. The tweezer can lift the sandwiched structure, indicating a strong coupling between hBN and the underlying substrate.

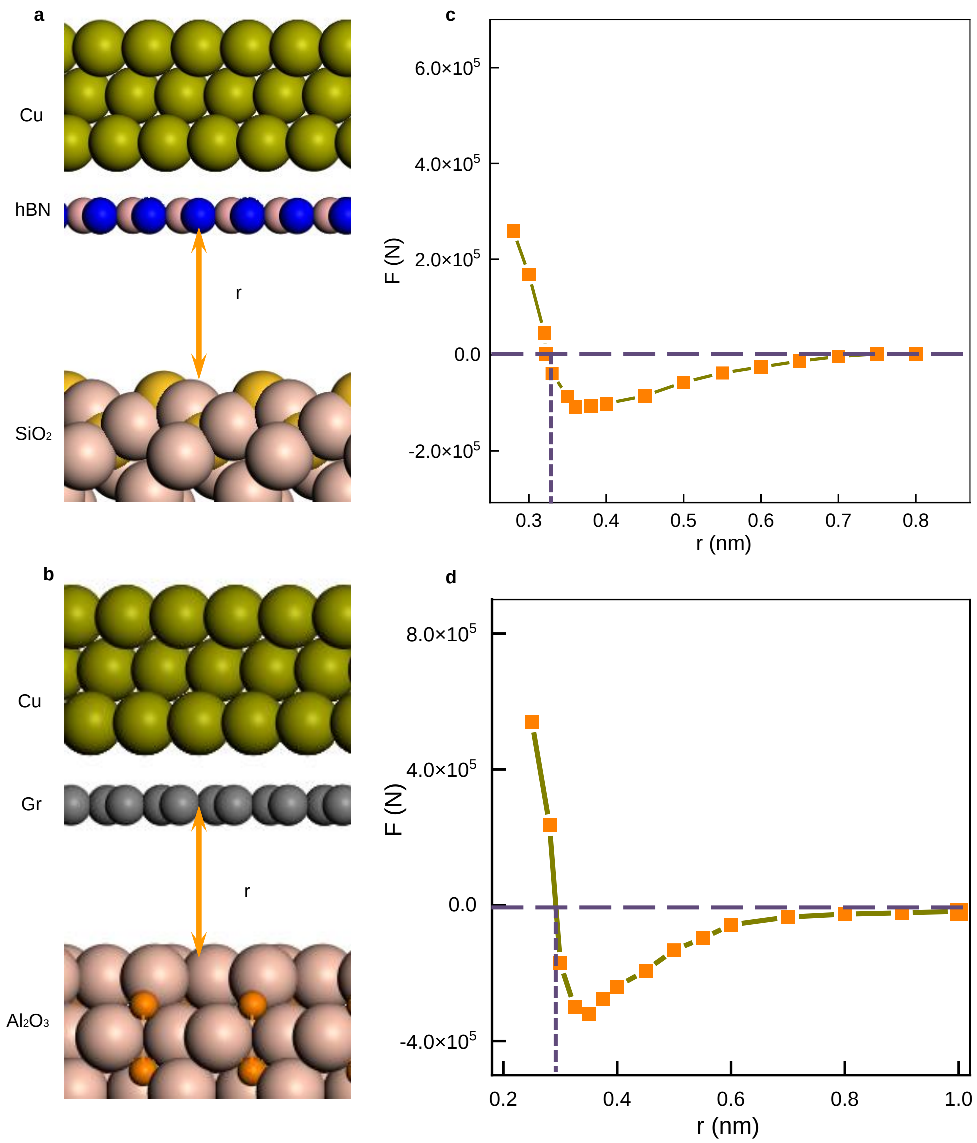

Supplementary Fig. 3 | Schematic diagrams of the Cu/hBN/SiO_2 (a) and Cu/graphene/Al_2O_3 structure (b). Plots and fit of the van der Waals force as a function of the distance between Cu/hBN/SiO_2 (c) and Cu/graphene/Al_2O_3 (d). For hBN, the attractive force is negligible when the distance is larger than 0.8 nm and increases with the decreasing of distance at the range of 0.33-0.8 nm. For hBN, the attractive force is negligible when the distance is larger than 1 nm and increases with the decreasing of distance at the range of 0.29-1 nm.

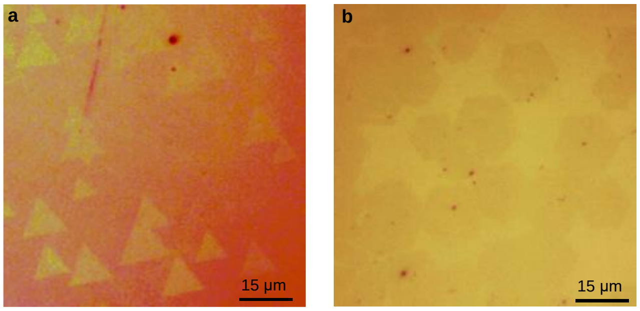

Supplementary Fig. 4 | Optical images of hBN (a) and graphene (b) taken from the SiO_2 side.

The images are adjusted to make the islands visible.

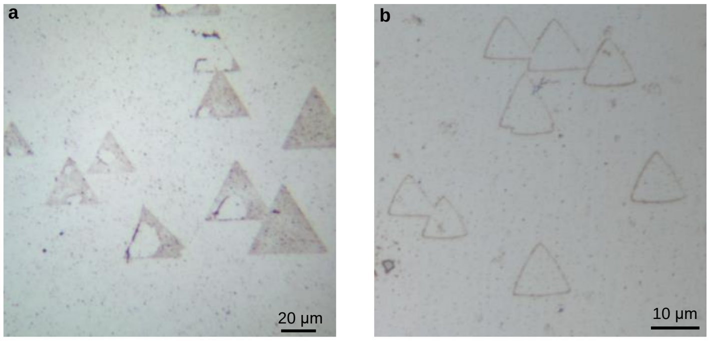

Supplementary Fig. 5 | Optical images of transferred hBN islands on SiO_2 substrate. Typical optical images of transferred hBN islands are shown in **(a-b)**. The transferred hBN islands are usually broken and dirty.

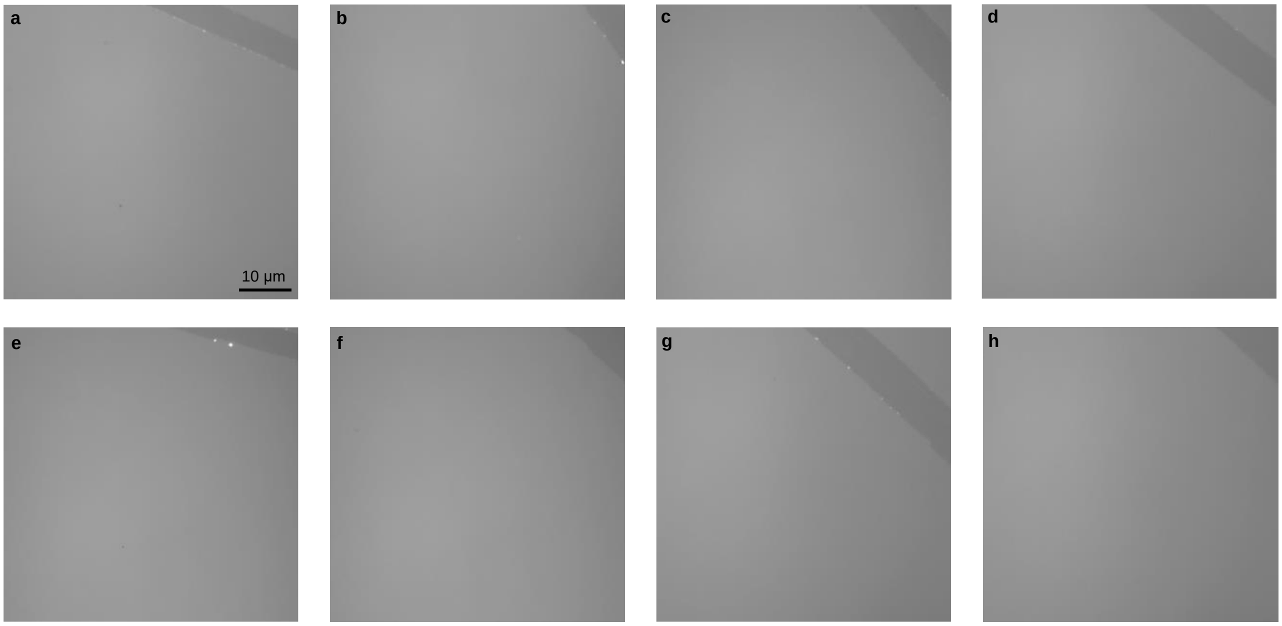

Supplementary Fig. 6 | Optical images of monolayer single-crystal hBN films on SiO_2 substrate at different positions. Scratches are deliberately made in the right corner to make the substrate and hBN samples distinguishable. The image sizes of a-h are same.

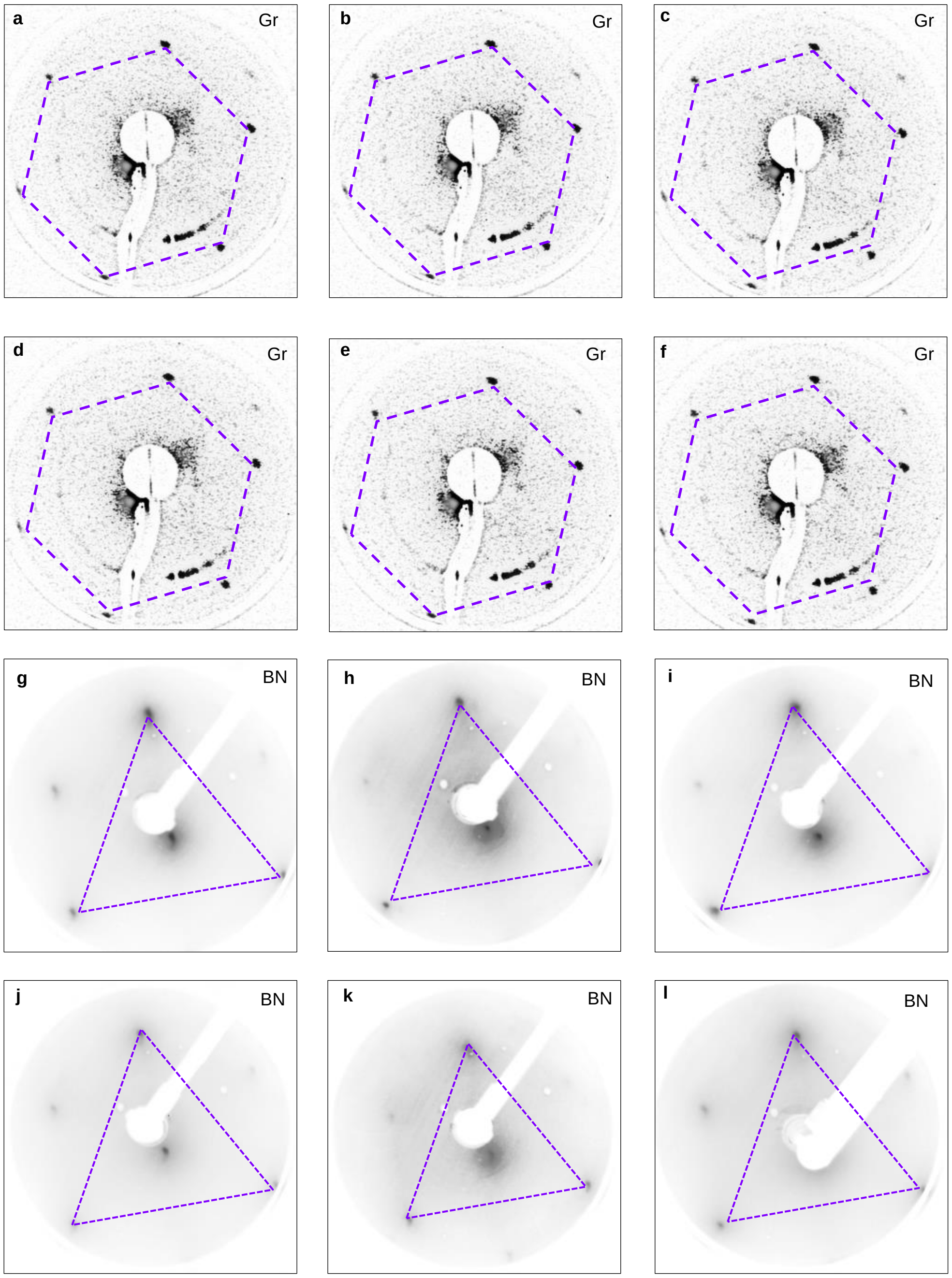

Supplementary Fig. 7 | LEED patterns of graphene (a-f) and hBN (g-l) samples at different areas. The identical orientations confirm the single-crystal nature.

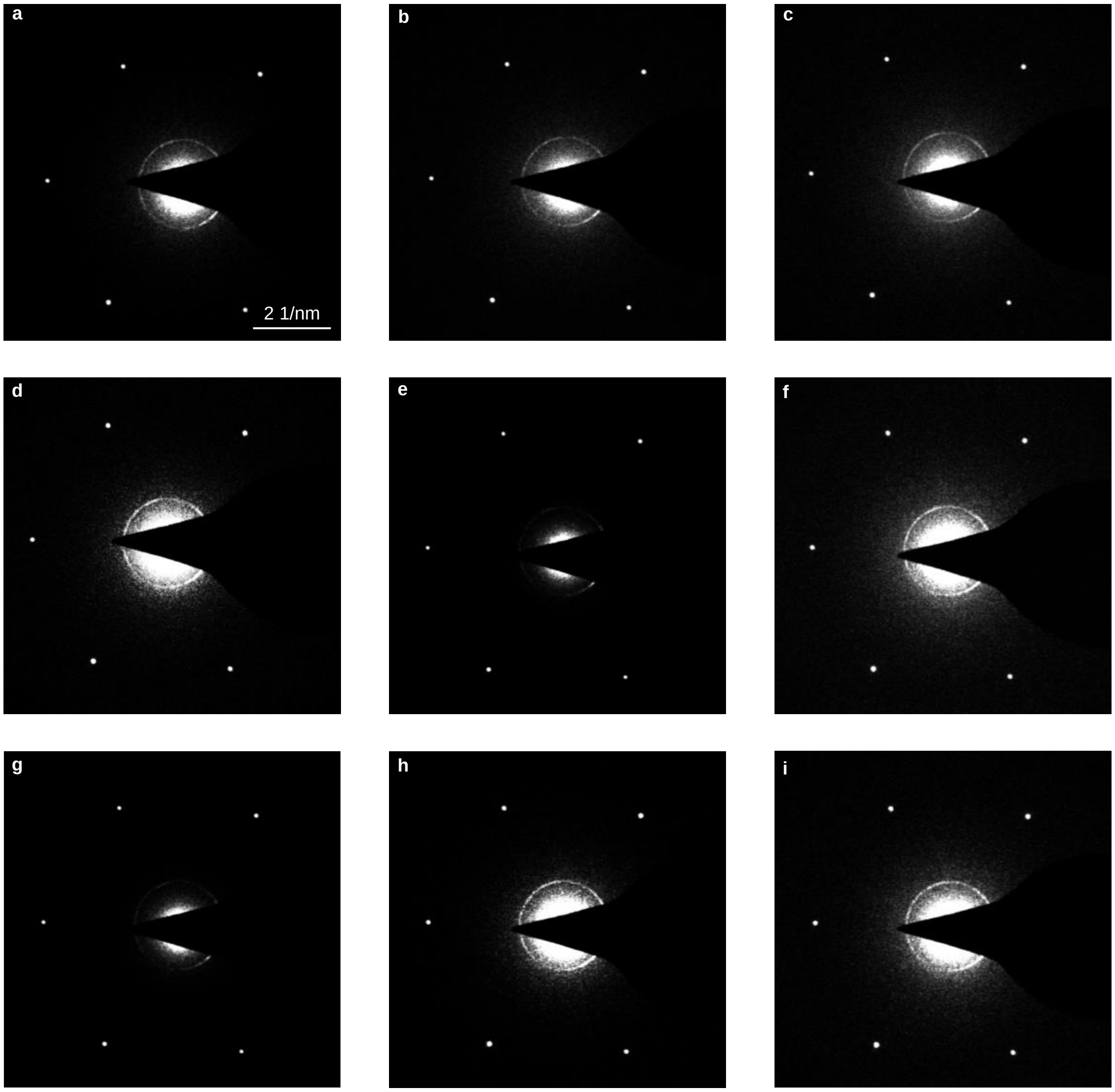

Supplementary Fig. 8 | Typical SAED patterns of as-grown hBN samples. The nearly identical crystallographic orientations reveal the single-crystal structure of the sample. The image sizes of **(a-i)** are same.

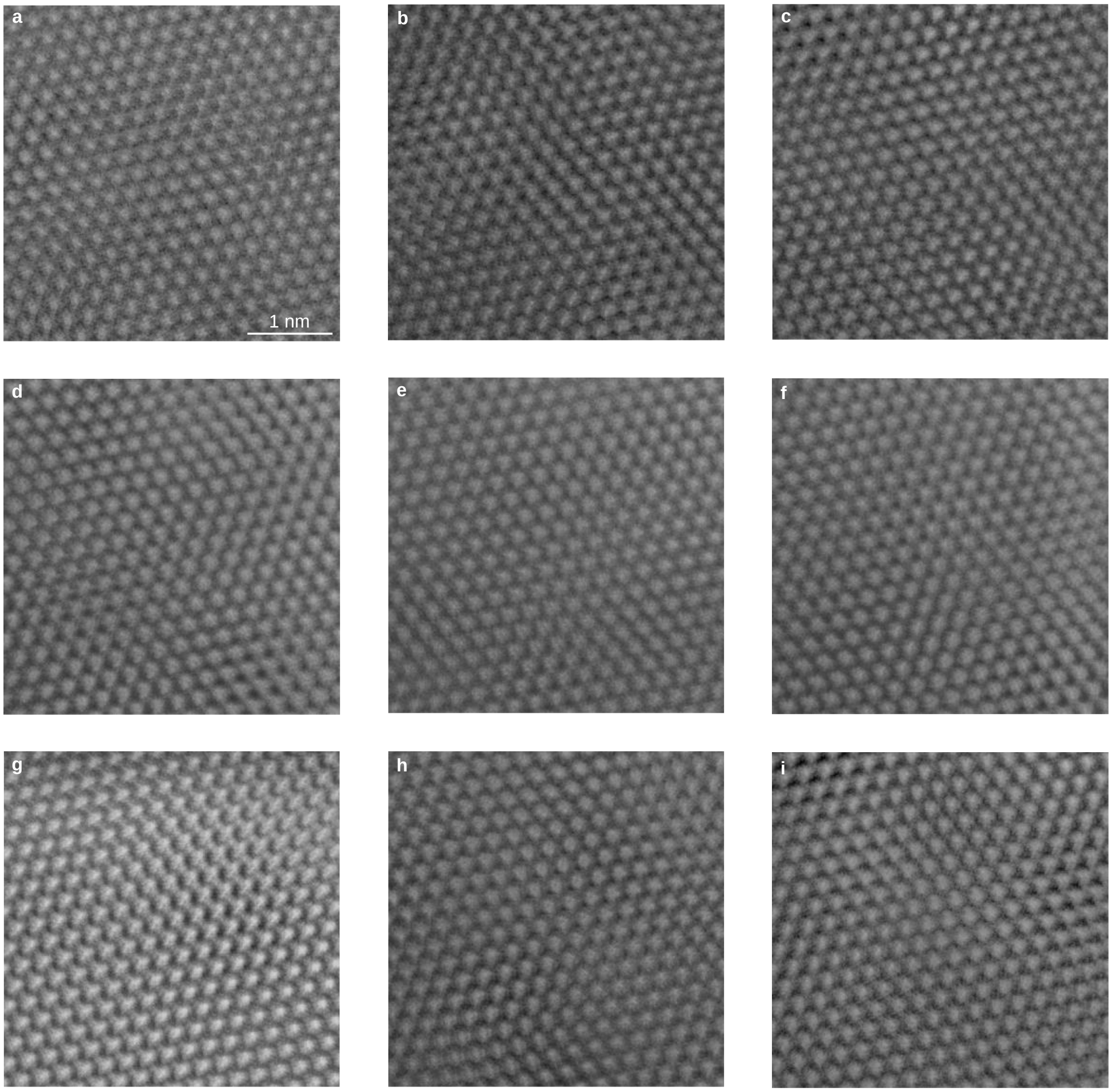

Supplementary Fig. 9 | Typical HRTEM images of as-grown hBN samples. The nearly identical lattice reveals the single-crystal structure of the sample. The image sizes of **(a-i)** are same.

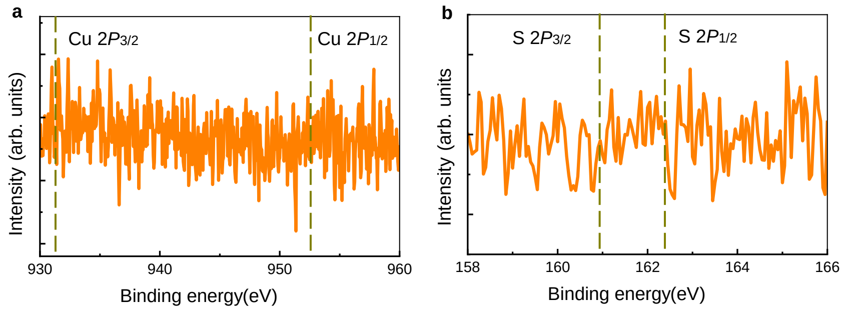

Supplementary Fig. 10 | XPS spectra of hBN samples on SiO_2 substrate. The absence of the peaks corresponding to Cu (a) and S (b) indicates no contamination of Cu or $(NH_4)_2S_2O_8$ on the hBN samples.

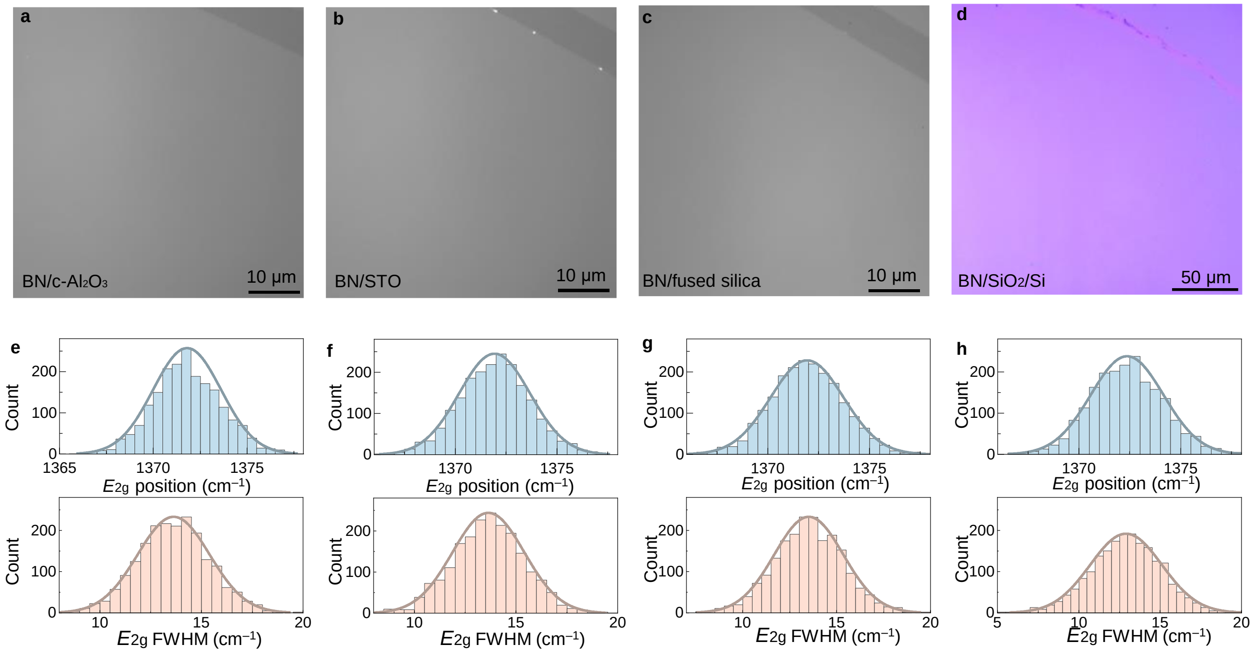

Supplementary Fig. 11 | Universal growth of hBN monolayers on various substrates. a-d, Optical image of hBN films grown on single-crystal c-plane sapphire **(a)**, $SrTiO_3(001)$ **(b)**, amorphous fused silica **(c)**, and SiO_2/Si **(d)** substrates. Scratches are deliberately made in the right corner to make the substrate and hBN/graphene samples distinguishable. **e-h,** Statistical distributions of the E_{2g}-band position and FWHM of hBN grown on c-plane sapphire **(e)**, $SrTiO_3(001)$ **(f)**, fused silica **(g)**, and SiO_2/Si **(h)** substrates.

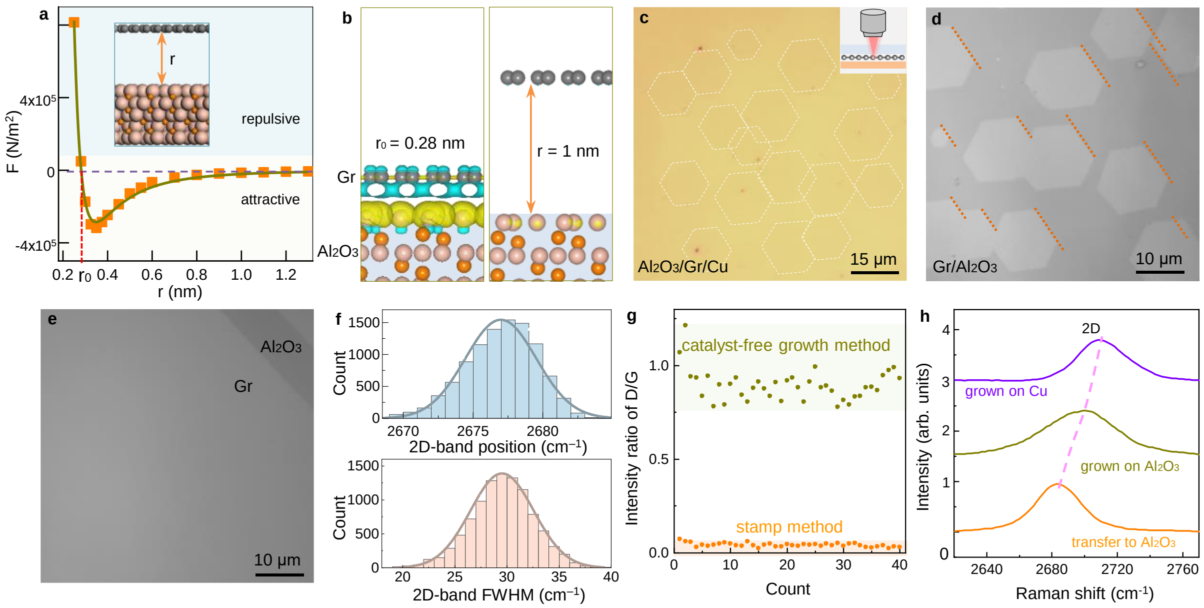

Supplementary Fig. 12 | Production of single-crystal graphene monolayer on Al_2O_3 substrate. a, Plot and fit of the van der Waals force as a function of the distance between graphene and Al_2O_3. The attractive force is negligible when the distance is larger than 1 nm and increased within the distance range of 0.28-1 nm. **b,** Charge density difference diagram of the graphene/Al_2O_3 with the distance of 1 nm and 0.28 nm. The yellow and blue colours correspond to the charge accumulation and depletion, respectively. **c,** Zoom-in optical image of Al_2O_3/graphene/Cu, aligned graphene islands can be seen as the Al_2O_3 substrate is transparent (the adjusted figure is shown in Supplementary Fig. 4b to make the islands clearer). Inset: schematic diagram of the light transmission. **d,** Optical image of as-grown graphene islands on Al_2O_3 after removing the Cu foil. **e,** Zoom-in optical image of as-grown graphene film. The top right corner in is an intentional scratch. **f,** Statistical distributions of the 2D-band position and FWHM of graphene. **g,** I_D/I_G ratio of 40 points of different samples prepared from catalyst-free growth method and stamp method. The green and orange colours correspond to catalyst-free growth method and stamp method, respectively. **h,** The 2D peak of Raman spectra of graphene grown on Cu (purple), grown on Al_2O_3 with our stamp method (green) and transferred to Al_2O_3 (orange).

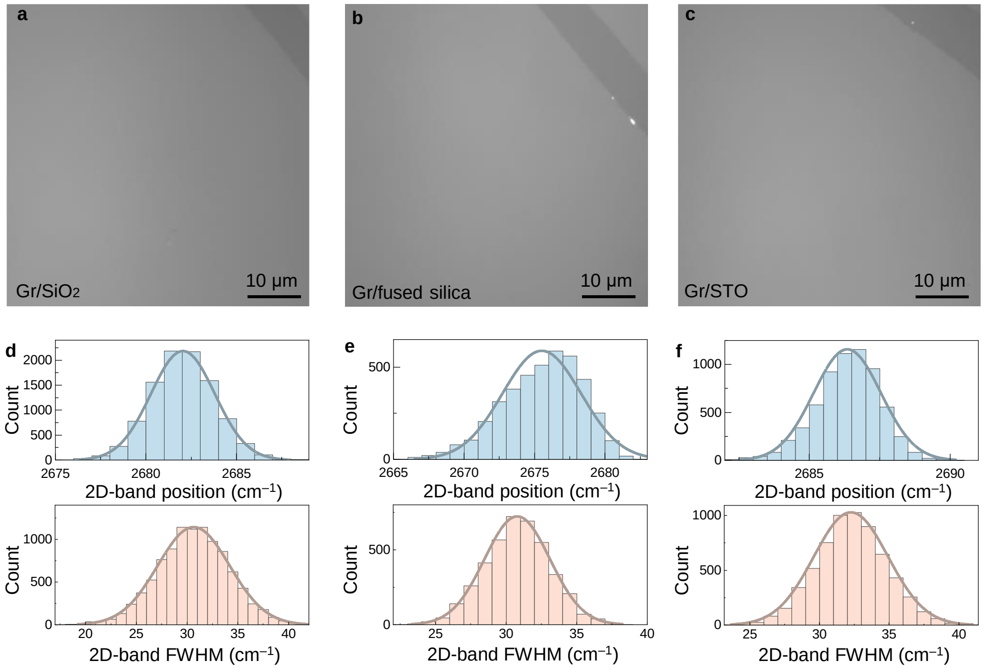

Supplementary Fig. 13 | Production of single-crystal graphene monolayer films on various insulating substrates. a-c, Optical image of as-grown graphene films on SiO_2 **(a)**, fused silica **(b)** and $SrTiO_3$ **(c)**. Scratches are deliberately made to make the substrate and graphene samples distinguishable. **d-f,** Statistical distributions of the 2D-band position and FWHM of graphene grown on SiO_2 **(d)**, fused silica **(e)** and $SrTiO_3$ **(f)** substrates.

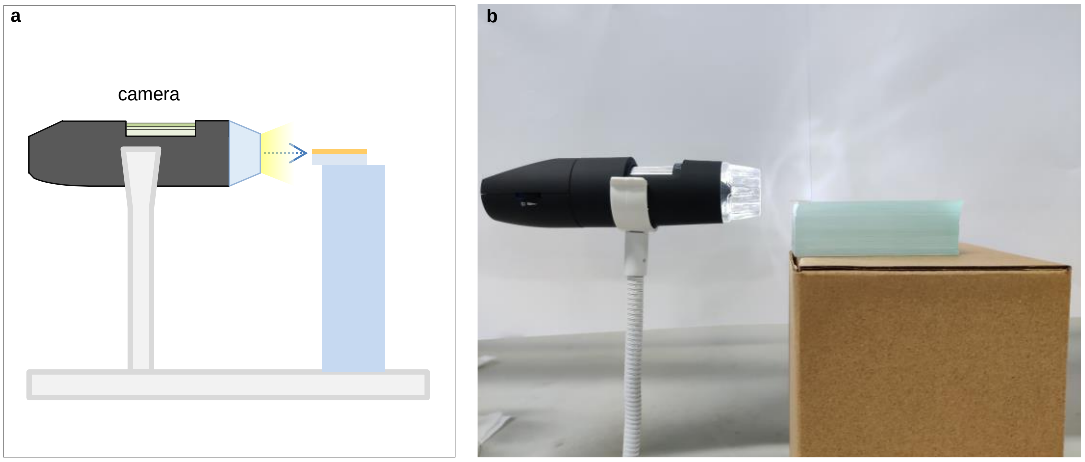

Supplementary Fig. 14 | Schematic diagram (a) and the real setup (b) of the camera to obtain the cross-sectional optical images.

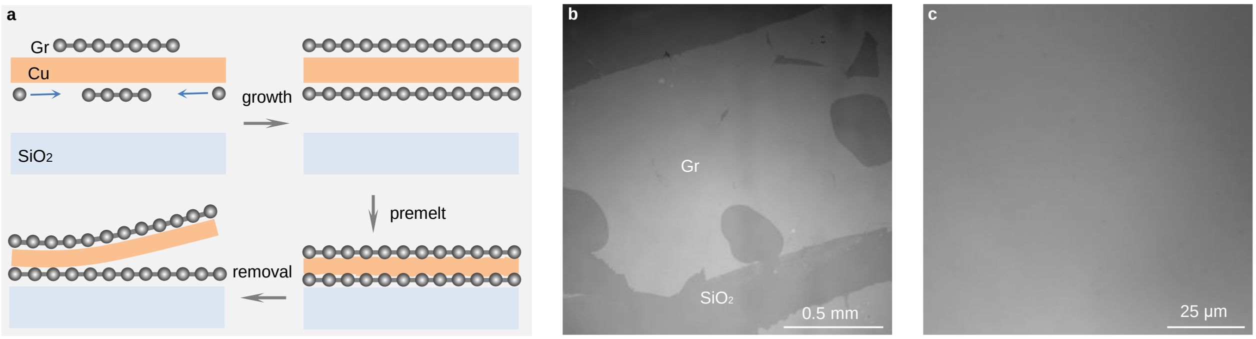

Supplementary Fig. 15 | a, Schematic diagram of the production of single-crystal graphene by directly peeling off the Cu foils. b-c, Optical images of graphene samples produced.
